# Dose painting based on tumor uptake of Cu-ATSM and FDG: a comparative study

**DOI:** 10.1186/s13014-014-0228-0

**Published:** 2014-10-16

**Authors:** Malene Martini Clausen, Anders Elias Hansen, Michael Lundemann, Christian Hollensen, Tobias Pommer, Per Munck af Rosenschöld, Annemarie Thuri Kristensen, Andreas Kjær, Fintan J McEvoy, Svend Aage Engelholm

**Affiliations:** Department of Oncology, Section of Radiotherapy, Rigshospitalet, University of Copenhagen, Copenhagen, Denmark; Department of Clinical Physiology, Nuclear Medicine & PET and Cluster for Molecular Imaging, Rigshospitalet, University of Copenhagen, Copenhagen, Denmark; Department of Veterinary Clinical and Animal Sciences, University of Copenhagen, Copenhagen, Denmark; Technical University of Denmark, DTU Nanotech, Center of Nanomedicine and theranostics, Lyngby, Denmark; Niels Bohr Institute, University of Copenhagen, Copenhagen, Denmark

**Keywords:** Hypoxia, Radiotherapy, Dose painting, Cu-ATSM, Positron emission tomography

## Abstract

**Background:**

Hypoxia and increased glycolytic activity of tumors are associated with poor prognosis. The purpose of this study was to investigate differences in radiotherapy (RT) dose painting based on the uptake of 2-deoxy-2-[^18^ F]-fluorodeoxyglucose (FDG) and the proposed hypoxia tracer, copper(II)diacetyl-bis(N^4^)-methylsemithiocarbazone (Cu-ATSM) using spontaneous clinical canine tumor models.

**Methods:**

Positron emission tomography/computed tomography scans of five spontaneous canine sarcomas and carcinomas were obtained; FDG on day 1 and ^64^Cu-ATSM on day 2 and 3 (approx. 3 and 24 hours pi.). Sub-volumes for dose escalation were defined by a threshold-based method for both tracers and five dose escalation levels were formed in each sub-volume. Volumetric modulated arc therapy plans were optimized based on the dose escalation regions for each scan for a total of three dose plans for each dog. The prescription dose for the GTV was 45 Gy (100%) and it was linearly escalated to a maximum of 150%. The correlations between dose painting plans were analyzed with construction of dose distribution density maps and quality volume histograms (QVH). Correlation between high-dose regions was investigated with Dice correlation coefficients.

**Results:**

Comparison of dose plans revealed varying degree of correlation between cases. Some cases displayed a separation of high-dose regions in the comparison of FDG vs. ^64^Cu-ATSM dose plans at both time points. Among the Dice correlation coefficients, the high dose regions showed the lowest degree of agreement, indicating potential benefit of using multiple tracers for dose painting. QVH analysis revealed that FDG-based dose painting plans adequately covered approximately 50% of the hypoxic regions.

**Conclusion:**

Radiotherapy plans optimized with the current approach for cut-off values and dose region definitions based on FDG, ^64^Cu-ATSM 3 h and 24 h uptake in canine tumors had different localization of the regional dose escalation levels. This indicates that ^64^Cu-ATSM at two different time-points and FDG provide different biological information that has to be taken into account when using the dose painting strategy in radiotherapy treatment planning.

## Background

Tumor hypoxia has been linked with radiation resistance since the 1950s, where Gray described a decreased radiosensitivity in oxygen-deprived cells [1]. Since then, several strategies for identification of hypoxic tumors have been proposed. Positron emission tomography (PET) holds a great promise for non-invasive detection of hypoxia, and various hypoxia specific tracers are investigated clinically [2-8]. Based on the Pasteur effect and its hypoxic induction of glycolytic genes, 2-deoxy-2-[^18^ F]-fluorodeoxyglucose (FDG) has also been suggested as a marker of hypoxia [9,10]. Elevated glycolytic activity and hypoxia are both associated with poor prognosis and are therefore potential targets for radiation dose escalation [3,4,11]. The concept of dose painting was introduced by Ling and colleagues, and is based on the idea that radio-therapeutic control can be improved by escalating the dose to tumor sub-volumes with known radio resistance identified with biological images [12]. The intratumoral distribution of glycolytic activity and hypoxia may be spatially determined by PET scans with FDG and the proposed hypoxia tracer, copper(II)diacetyl-bis(N^4^)-methylsemithiocarbazone (Cu-ATSM) [10,13,14].

Previous studies in human tumors and experimental xenografts have identified a potential overlap between hypoxic regions and glycolytic activity. However, the results have been varying and the link between FDG uptake and tumor hypoxia has not been consistent [15-21]. Hypoxia induces an up-regulation of glycolytic activity, but an increased growth rate also increases glycolytic activity and thus increasing the likeliness of regional cellular expansion beyond the capability of neoangiogenesis, thus inducing regional hypoxia [22,23]. Situations may exist where the basic glycolytic activity (Warburg effect) of the tumor may exceed the capability of hypoxia to induce additional glycolysis (Pasteur effect). The dynamics of tumor hypoxia can also explain some of the varying results. Tumor hypoxia occurs as both chronic hypoxia and acute hypoxia [23], and may therefore influence the regional overlap of hypoxia and glycolytic activity. Acute perfusion limited hypoxia may induce cycling hypoxic changes which may not be validly appreciated by using glycolytic activity as a marker of hypoxia. Additionally, cycling hypoxic changes may induce situations where changes in glycolytic activity may be trailing [24,25].

Dose painting by contours (DPBC) refers to dose escalation to a threshold-defined sub-volume within the tumor. It is an attractive strategy since it is clinically feasible and standard commercial treatment planning software can be used [26]. In a previous study we investigated the overlap between sub-volumes of ^64^Cu-ATSM and FDG uptake in a DPBC approach. We showed that sub-volumes displayed a varying degree of overlap, but that FDG by this strategy is not a marker of hypoxia, as defined with ^64^Cu-ATSM, in RT planning [27]. Given that both FDG and ^64^Cu-ATSM are negative prognostic factors for RT [3,4,28,29], combining the two radiotracers in treatment planning seems attractive. However, including multiple tracers in a biological target volume (BTV) only allows a limited dose escalation as the BTV then represents a large fraction of the gross tumor volume (GTV). In order to target both tracers, a more sophisticated dose prescription that allows a highly conformal dose delivery with possibilities for redistribution of dose is required. Bentzen proposed a new aspect of dose painting by the introduction of “dose painting by numbers” (DPBN) in 2005 [30]. This strategy accommodates the problem of binary volumes in DPBC, and allows a linear relationship between PET uptake and prescribed dose, which is biologically more intuitive and where higher peak doses can be achieved [26]. However, the voxel-based treatment planning is complex and special software is required. Planning and delivery of a complex dose painting case has previously been proven feasible using volumetric modulated arc therapy (VMAT), (RapidArc, Varian Medical Systems, Palo Alto, US) [31].

This study investigates the influence of the correlation between glycolytic activity and hypoxia for RT planning in spontaneous canine tumors. Based on our previous findings it seems attractive to include the intersection volume between FDG and ^64^Cu-ATSM in the target volume. The intersection volume constitutes a relatively small fraction of the total GTV and thus allows a substantial dose increase compared to the BTV [27]. However, this strategy is only valuable if high-uptake regions for FDG and ^64^Cu-ATSM are co-localized within the intersection volume.

The aim of this study is to evaluate and compare RT dose painting plans based on a DPBN approach for PET scans of FDG and ^64^Cu-ATSM at two different time points post injection. Standard commercial treatment planning software is used for this dose escalation strategy where five dose escalation levels are defined from PET uptake within the GTV.

Furthermore, we investigate if a dose painting plan based on FDG-uptake can replace a hypoxia-guided ^64^Cu-ATSM-based dose painting plan by analysis of quality volume histograms (QVH).

## Methods

### Tumor imaging and image reconstruction

PET/CT data from five spontaneous canine cancer patients with sarcomas or carcinomas were included in this planning study in accordance with a previously described protocol approved by the local ethics committee [32]. All dogs were scanned with baseline FDG PET/CT on day 1 (approx. 1 h p.i.) and ^64^Cu-ATSM PET/CT on day 2 and 3, approx. 3 h and 24 h p.i. A mean activity of 7.7 MBq/kg was injected for both tracers. Dogs were anaesthetized and immobilized during the scanning procedure to ensure comparable images. Scanning and reconstruction parameters were described in a previous study [27].

### Target volume definition

PET/CT images were transferred to Eclipse v10 treatment planning software (Varian Medical Systems, Palo Alto, CA, US). The gross tumor volume (GTV) was delineated by a veterinarian and an experienced radiologist and on the day 1 CT. Images were manually co-registered in Image Registration (Varian Medical Systems, Palo Alto, CA, US) with 3 h ^64^Cu-ATSM (day 2) as the reference image.

The high-risk sub-volume of each tumor was defined by thresholding of standardized uptake values (SUV) in a DPBC approach as previously described [27]. The cut off value for FDG was 40% of SUV_max_ [33], whereas the 3 h ^64^Cu-ATSM sub-volume (Cu3) was defined by an absolute SUV of 1.4, reflecting tumor hypoxia defined by an oxygen tension below 10 mmHg [34].

No previous studies have defined a cut off value for long distribution times of ^64^Cu-ATSM, and tumor-to-muscle ratio was therefore used for calculation of the cut off value for the 24 h ^64^Cu-ATSM (Cu24) sub-volumes. In order to reach a higher peak dose, the dose prescription was based on a DPBN related method and five dose escalation levels were defined within the high-risk sub-volume. For each dose level, a target substructure was created in Eclipse for dose optimization (DP1-DP5 from low to high dose). The segmentation of PET images was performed at a Leonardo Workstation (Siemens, Erlangen, Germany), and dose levels were defined based on the assumption that PET uptake and radiosensitivity display a linear association.

### Dose prescription

A DPBN approach with a linear escalation to the target was used for dose prescription. However, due to the segmentation into five dose levels, dose delivery is only an approximation of this linear relationship. At our institution the standard pre-operative RT protocol for canine cancer patients with soft tissue sarcoma is 4.5 Gy in 10 fractions, 3 fractions per week, which was used for dose prescription in this planning study. The GTV received 100% of the prescribed dose, and radiation dose was escalated to the five dose levels with the high-uptake region receiving a maximum of 150%. This dose escalation has previously been associated with an increased tumor control probability (TCP) [35].

The first dose escalation level for each PET scan was determined by a DPBC cut-off value as mentioned above. The cut-off values were either a percentage of SUV_max_ (FDG) or an absolute uptake value (^64^Cu-ATSM). All values for the first dose levels (DP1) were calculated as a percentage of the maximum uptake given as *‘SUV*_*c*_*’* in the dose prescription equation beneath. The dose for each dose level was calculated based on the mean value between cut-off values for two neighboring dose levels, *‘SUV*_*iso*_*’*:

$$ Dose\ \left(\%\right)=100+\frac{SUV\ iso - SUV\ c\ }{100 - SUV\ c}\times 50, $$

### Treatment planning

RapidArc plans using 6 MV photons and a multi leaf collimator with 2.5 mm leaf width were optimized based on the dose escalation regions for each tracer for a total of three dose plans for each dog. All treatment plans were constructed with two arcs and complementary collimator rotation angles of 45 and 315 degrees were used. The dose plan was optimized with upper and lower dose limits for each target substructure, securing the calculated dose for the respective dose level. All substructures were prioritized equally and no dose-constraints were applied for organs at risk. For accurate modeling of dose deposition in the heterogeneous target with small dose regions, the anisotropic analytical algorithm (AAA) was used as calculation model with a 1 mm grid.

### Correlation of dose painting plans

For all GTVs, the dose in Gy per voxel was analyzed in MATLAB (R2009b, MathWorks, Natick, MA, US) and treatment plans based on FDG and ^64^Cu-ATSM at 3 h and 24 h p.i. were compared based on 2D histograms.

To analyze whether an FDG-based dose painting plan can sufficiently cover tumor hypoxia assessed by the uptake of ^64^Cu-ATSM, QVH between FDG and ^64^Cu-ATSM plans were constructed. QVHs resemble the dose volume histograms and are in this study obtained by calculation of the ratio between the planned dose for FDG and the planned dose for ^64^Cu-ATSM at both time points. The QVH reflects the plan conformity, and a steep curve with a quality index (QI) of 1 resembled a perfect match between the two plans; a value between 0.95 and 1.05 was considered satisfactory. QVHs were produced for the entire GTV in order to assess the quality of the following comparisons of plans: FDG vs. Cu3, FDG vs. Cu24 and Cu3 vs. Cu24. The latter QVH was assessed due to the previously described temporal differences in the uptake of ^64^Cu-ATSM [32]. The co-localization of high-dose regions in all dose painting plans was analyzed by calculation of the Dice correlation coefficient for each dose level.

## Results

Patient characteristics, sub-volume sizes and SUV are given in Table 1. The mean SUV for Cu3 was 2.8 (range: 2.3-3.6) and the cut off for Cu3 at SUV 1.4 therefore corresponds to an approximated 50% of SUV_max_, which is a frequently used threshold for other PET tracers. Dose prescription was calculated based on PET-uptake as described above, and the following mean doses (range) were prescribed to dose levels: DP1 47.3 Gy (47.1-47.5), DP2 51.8 Gy (51.5-52.0), DP3 56.3 Gy (56.1-56.3), DP4 60.7 Gy (60.3-60.8) and DP5 65.3 Gy (65.2-65.3). Table 2 shows the mean volumes of all dose levels for FDG, Cu3 and Cu24.

**Table 1:** **Tumor characteristics, sub-volume data and SUV**

**Dog no.**	**Tumor type**	**Tumor localization**	**GTV (ccm)**	**FDG (ccm)**	**Cu3 (ccm)**	**Cu24 (ccm)**	**SUV**_**max**_ **FDG**	**SUV**_**max**_ **Cu3**
1	Hemangiopericytoma	Lumbar region	128.7	13.0	85.8	61.6	7.9	2.3
2	Fibrosarcoma	Lat. cervical region	88.2	66.4	63.9	17.0	8.5	3.6
3	Squamous cell carcinoma	Nasal cavity	55.4	23.7	21.2	30.0	23.1	3.0
4	Adenocarcinoma	Nasopharynx	20.8	10.3	9.3	3.5	13.2	2.5
5	Undifferentiated soft tissue sarcoma	Mandible	23.2	11.2	6.2	0.6	9.7	2.6
Avg.			63.3	24.9	37.3	22.5	12.5	2.8

**Table 2:** **Dose level volumes (mean values)**

**Dose level**	**FDG (ccm)**	**Cu3 (ccm)**	**Cu24 (ccm)**
DP1	8.56	12.69	7.73
DP2	6.02	8.51	3.03
DP3	4.14	3.45	0.61
DP4	1.64	1.13	0.09
DP5	0.19	0.17	0.00

In six treatment plans, the highest dose level (DP5) could not be transferred to the treatment planning system, since the substructure was present at only one slice of the CT scan and therefore not a potential volume for VMAT optimization. The DP5 substructure was lost in four out of five cases for the Cu24 dose plan and in two cases for Cu3. None of the FDG substructures were lost in the transfer to treatment planning. The transfer of contours and volumes between software caused volumetric estimates to be somewhat dissimilar (Tables 1 and 2). All contours were visually inspected in both Leonardo Workstation and Eclipse, and the geometrical differences were found to be inconsequential for the purpose of dose painting and treatment planning.

**Figure 1:**
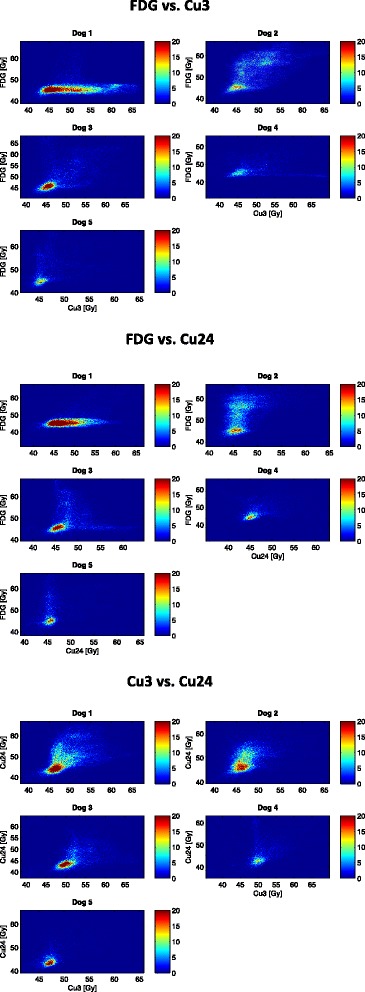
**2D histograms for comparison of dose painting plans.** A comparison of FDG and ^64^Cu-ATSM dose painting plans showing diverging results. The FDG and Cu3 2D histograms tend to display a separation of the high-dose areas indicating an increasing mismatch at the high-dose levels and therefore a poor degree of overlap between the dose plans. The dose is distributed with an even greater variation when comparing FDG and Cu24. The two dose painting plans based on early and late ^64^Cu-ATSM demonstrate an improved correlation compared to FDG vs. ^64^Cu-ATSM, however linearity is not observed.

The comparison of dose painting plans was based on the assumption that the GTV did not change anatomically in between the scans. The 2D histograms in Figure 1 illustrate a large variation in the correlation of dose painting plans for each dog. There is a general trend for a more linear correlation between Cu3 and Cu24, whereas FDG vs. Cu3 and Cu24 in some cases show a distinct separation of the high-dose areas. In all cases, a high intensity of dose is observed at 45 Gy, which is the base dose delivered to the total GTV. Correlations of PET-uptake within the total GTV are similar to the 2D histograms (data not shown).

**Figure 2:**
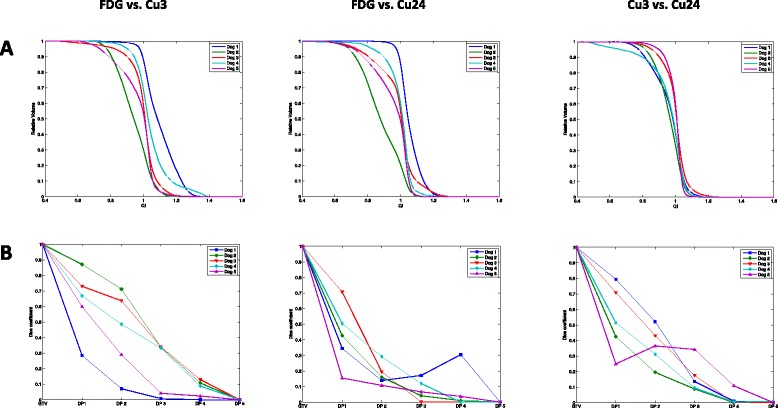
**QVH (A) and Dice correlation coefficient (B).** Comparison of dose painting plans: FDG vs. Cu3, FDG vs. Cu24 and Cu3 vs. Cu24. The Dice correlation coefficient decreases with increasing dose level, and no correlation is observed for the high-dose regions.

QVH analysis to assess the feasibility of covering a hypoxia-guided target based on FDG dose painting is depicted in Figure 2A. In some cases it is possible to achieve an acceptable coverage for a relatively large percentage of voxels. As described above, the treatment plan was considered satisfactory with a QI between 0.95 and 1.05. This is obtained for approximately 50% of voxels for comparisons of FDG and ^64^Cu-ATSM, whereas the association between Cu3 vs. Cu24 displays a better coverage with 60% of the voxels having a sufficient QI (Table 3).

**Table 3:** **The fraction of voxels with an acceptable QI**

**Dog no.**	**FDG vs. Cu3**	**FDG vs. Cu24**	**Cu3 vs. Cu24**
1	0.40	0.57	0.53
2	0.34	0.27	0.48
3	0.64	0.56	0.67
4	0.52	0.68	0.56
5	0.58	0.56	0.80
Mean	0.49	0.53	0.61

Figure 2B illustrates Dice correlation coefficients for each dose level of FDG vs. Cu3 and Cu24. It shows variation between cases, but a clear trend for an increasing mismatch between dose painting plans with higher dose levels is observed. In general, the steepness of the coefficient drop is greater for FDG vs. Cu24. Correlation coefficients for the comparison of Cu3 and Cu24 also display varying results between cases. The probability for correlation between dose levels reflected by Dice correlation coefficients naturally decreases with decreasing volume of dose levels.

## Discussion

In this study, highly heterogeneous dose painting plans based on FDG and ^64^Cu-ATSM were compared in order to assess whether an FDG-based treatment plan could sufficiently cover tumor hypoxia and thereby replace ^64^Cu-ATSM based dose painting.

Comparison of dose plans using 2D histograms revealed a mismatch between treatment plans based on FDG and ^64^Cu-ATSM (3h and 24h pi.). Some cases displayed a correlation pattern indicating no overlap between the high-dose regions. The high-dose regions showed the lowest degree of agreement, indicating that inclusion of combined information from multiple tracers for dose painting may improve therapeutic benefit. These findings were primarily observed in the correlation between FDG and ^64^Cu-ATSM treatment plans at both time points. There was a tendency towards a stronger correlation between Cu3 and Cu24 plans, suggesting that ^64^Cu-ATSM distribution time is less important than the PET tracer used for phenotypical characterization of the tumor.

Previous studies have compared the overlap between FDG-uptake and hypoxia by imaging comparisons, autoradiography and gene expression analyses with inconclusive results [10,15-17,20,32,36]. Including tumor physiologic aspects assessed by PET in treatment planning seems attractive, and may potentially lead to improved tumor control if a clinically feasible and a reproducible technique is established. Validation of the proposed PET tracers including the combined information is the first step of this process, while clarifying how the PET signal is translated into RT target volumes in a clinically relevant and robust approach is the second step. Considering the scale on which changes in the tumor microenvironment occur, the spatial resolution of the PET system is poor. One voxel may represent a wide variety of oxygen tensions, and converting the PET signal from numerous voxels within a tumor to dose painting targets will inevitably lead to loss of information [37-39]. In regards to the overlap between FDG and ^64^Cu-ATSM, the question is whether this loss of information results in a smoothing of differences or even larger mismatches between the tracers. A heterogeneous uptake of both PET tracers may indicate that hypoxic regions are missed when treatment optimization is based on FDG only. In this study, PET images and 2D histograms displayed similar correlation patterns indicating that treatment planning based on defined dose levels does not cause significant loss of PET information.

FDG is the most frequently clinically used PET tracer and high uptake is associated with several malignant characteristics including increased growth rate and hypoxia [10]. It therefore seems attractive to include only FDG-avid regions in RT planning. However, the strong prognostic effect of tumor hypoxia and the increased evidence that FDG uptake does not sufficiently include hypoxia makes multi-tracer RT planning attractive. In this study, we explored the possibilities for applying an FDG-based dose-painting plan on tumor hypoxia assessed by the uptake of ^64^Cu-ATSM. QVH analysis revealed that an acceptable coverage of hypoxic regions could be obtained for only approximately 50% of the voxels emphasizing the need for alternative strategies in hypoxia-guided RT.

This is to our knowledge the first study to compare dose painting treatment plans based on two PET-tracers. In a previous study on the same group of canine cancer patients, we observed an overlap between FDG and ^64^Cu-ATSM based on a DPBC approach [27]. However, dose escalation to a target volume including both PET tracers in a BTV was not feasible, as the volumes represented a large fraction of the GTV and therefore could lead to increased toxicity. Targeting the intersection volume between FDG and ^64^Cu-ATSM would allow inclusion of both tracers for DPBC treatment planning. The feasibility of increasing tumor control by this strategy requires co-localization of high-dose regions within the intersection volume. Calculation of Dice correlation coefficients revealed an increasing mismatch between FDG and ^64^Cu-ATSM dose painting plans; the higher dose region, the larger mismatch. However, these data must be carefully interpreted as the very small high-dose regions lower the likelihood of an intersection volume to exist. A poor correlation between the high-dose regions of FDG and ^64^Cu-ATSM can still allow the targeting of both tracers in a DPBC approach, as the small volumes may be co-localized within the intersection volume. Furthermore, it is important to mention the influence of the chosen thresholds in this study. The highest dose level, DP5, could not be transferred to the dose planning software in four of five cases for Cu24, and the structure was also lost in two cases for Cu3. If a different threshold had been chosen, these results might have differed. However, the trend for a lower degree of correlation between the high dose areas seems clear.

The dose level volumes of Cu24 are generally smaller than those of FDG and Cu3, which may explain the poorer correlation between FDG and Cu24.

The delivery of heterogeneous dose distribution with standard treatment planning software has been proven technically feasible [31] and modeling studies also suggest that biologically adapted RT can lead to large improvements in TCP [40-43]. However, several open and unanswered questions still need to be clarified before clinical implementation. The clinical feasibility of hypoxia-guided dose painting is highly dependent on a robust target throughout the treatment course as a mismatch between delivered dose and hypoxic regions potentially result in decreased tumor control. Tumor hypoxia is known to be a dynamic and clinical observations of tumor reoxygenation suggest that the cycling changes can occur within minutes, hours or even days [39]. It has been shown that relatively large regions within tumors exhibit transient periods of hypoxia and reoxygenation [44], which is also reflected by the studies of pretreatment tumor hypoxia reproducibility [45,46]. Voxel-by-voxel correlations previously done on this series of PET/CT scans displayed temporal changes in the uptake of ^64^Cu-ATSM suggesting that hypoxia-guided dose painting treatment plans may vary markedly dependent on time point [32]. Hypoxia-guided dose painting therefore requires further evaluation of the temporal stability of tumor hypoxia and the need for adapted treatment during the course of RT. The delivery of highly heterogeneous dose distributions also demands tumor movement to be taken into account; especially in this DPBN based strategy with complex and often small target volumes where margins cannot be added.

There are several drawbacks to this planning study including a low number of cases with varying histopathologies. In our study, no trend was observed between histopathology and the correlation of FDG and ^64^Cu –ATSM, however, in a larger material, a difference between sarcomas and carcinomas may be observed. The segmentation method and the threshold-based treatment planning may also influence the observed correlations between FDG and ^64^Cu-ATSM dose painting plans; especially the thresholding of late ^64^Cu-ATSM PET must cautiously interpreted as no previous studies have described a cut-off value for tumor hypoxia at 24 p.i. As mentioned above, the Dice correlation coefficients are affected by the size of the analyzed volumes and poorer correlations are therefore expected for small volumes, and co-localization of high-dose regions in the intersection volume cannot be excluded.

This study must be considered exploratory due to the drawbacks mentioned above, including the thresholding, which may influence the obtained results. The low degree of overlap between high-dose regions may therefore be explained by the chosen threshold procedure, and further studies are needed in order to draw any firm conclusions.

## Conclusion

The correlation analysis of dose plans based on FDG, ^64^Cu-ATSM 3 h and 24 h uptake in canine tumors showed that regional dose escalation levels have different localizations based on the current approach for cut-off values and dose region definition. This indicates that ^64^Cu-ATSM at two different time-points and FDG provide different biological information that has to be taken into account when applying the dose painting strategy in RT planning. Based on current knowledge with poor prognosis being related to both hyperglycolysis and hypoxia and the result from this study where only a partial overlap is observed between FDG and ^64^Cu-ATSM, the inclusion of multiple tracers in RT planning seems attractive. In such strategy, a number of boost levels within each high-risk sub-volume will be the most attractive approach in order to improve tumor control as the localization of high-uptake regions are different for the two tracers.
